# A Systematic Stereo Camera Calibration Strategy: Leveraging Latin Hypercube Sampling and 2^k^ Full-Factorial Design of Experiment Methods

**DOI:** 10.3390/s23198240

**Published:** 2023-10-03

**Authors:** Yanan Hao, Vin Cent Tai, Yong Chai Tan

**Affiliations:** 1 Department of Electronic Engineering, Taiyuan Institute of Technology, Taiyuan 030008, China; yananhao2020@163.com; 2 Faculty of Engineering, Built Environment and Information Technology, SEGI University, Petaling Jaya 47810, Malaysia; taivincent@segi.edu.my

**Keywords:** camera calibration, binocular camera, reprojection error, Latin Hypercube Sampling, design of experiment

## Abstract

This research aimed to optimize the camera calibration process by identifying the optimal distance and angle for capturing checkered board images, with a specific focus on understanding the factors that influence the reprojection error ($\epsilon_{RP}$). The objective was to improve calibration efficiency by exploring the impacts of distance and orientation factors and the feasibility of independently manipulating these factors. The study employed Zhang’s camera calibration method, along with the 2^k^ full-factorial analysis method and the Latin Hypercube Sampling (LHS) method, to identify the optimal calibration parameters. Three calibration methods were devised: calibration with distance factors (D, H, V), orientation factors (R, P, Y), and the combined two influential factors from both sets of factors. The calibration study was carried out with three different stereo cameras. The results indicate that D is the most influential factor, while H and V are nearly equally influential for method A; P and R are the two most influential orientation factors for method B. Compared to Zhang’s method alone, on average, methods A, B, and C reduce $\epsilon_{RP}$ by 25%, 24%, and 34%, respectively. However, method C requires about 10% more calibration images than methods A and B combined. For applications where lower value of $\epsilon_{RP}$ is required, method C is recommended. This study provides valuable insights into the factors affecting $\epsilon_{RP}$ in calibration processes. The proposed methods can be used to improve the calibration accuracy for stereo cameras for the applications in object detection and ranging. The findings expand our understanding of camera calibration, particularly the influence of distance and orientation factors, making significant contributions to camera calibration procedures.

## 1. Introduction

Binocular or stereo vision, used in diverse areas like autonomous driving [1,2], robot navigation [3,4], 3D reconstruction [5,6,7], and industrial inspection [8,9], employs two identical cameras to capture two images of the same target from different vantage points. The disparity between these images enables 3D reconstruction and measurement, the accuracy of which is heavily dependent on the precision of the camera calibration and stereo matching methods, thus making these processes key research subjects in stereo vision. During the camera calibration process, it is crucial to estimate both the intrinsic parameters, such as the focal length and optical centers, and the extrinsic parameters, like the camera’s position and orientation in space. These directly influence the calculation of 3D spatial information [10]. Camera calibration significantly impacts the accuracy of the reconstruction and measurement, thereby playing a crucial role in binocular vision.

Camera calibration techniques mainly fall into two categories: self-calibration and photogrammetric calibration [11]. Self-calibration methods, though not needing a calibration object, rely on sequences of images from an uncalibrated scene and often entail complex computations [12,13,14] and low in accuracy [5,15]. Conversely, photogrammetric methods, including the popular methods such as Zhang’s [16], Tsai’s [17], Bouguet’s [18], and Heikkila and Silven’s [19] methods utilize geometric information from a calibration object, such as a checkered board with known geometry, to determine camera parameters. The latter methods minimize $\epsilon_{RP}$ through feature point detection and optimization, providing more accurate information about camera characteristics.

Zhang’s camera calibration method [16] has gained significant attention due to its simplicity, robustness, and high degree of accuracy. One of the key advantages of Zhang’s method is that it only requires a simple calibration object, such as a checkered board, which is easy to fabricate and measure. This stands in contrast to other photogrammetric methods that may require specialized calibration objects or more complicated patterns. The method is based on the fundamental matrix theory, which determines the intrinsic (i.e., focal length, lens distortion coefficients, and the position of the image center) and extrinsic (i.e., camera position and orientation) parameters of a camera by measuring the geometric relationship between the camera and the calibration board. The method requires at least three pairs of images of a checkered board with known dimensions taken from different positions and orientations within the camera’s field of view (FOV). Then, a corner detection algorithm is employed to detect the corners in each image, i.e., the intersections between the black and white squares, and the image coordinates of the detected corners are matched with their known 3D coordinates in the checkered board. Subsequently, least-squares minimization, i.e., the Levenberg–Marquardt algorithm, is used to search for determining the camera’s intrinsic and extrinsic parameters that yield minimum geometric error between the projected 3D calibration points and their corresponding 2D image points. The lens distortion coefficients, such as radial and tangential distortions, are also estimated and corrected.

Owing to the simplicity, flexibility and robustness of Zhang’s method, many researchers have developed new camera calibration algorithms that incorporate Zhang’s method to improve calibration accuracy. Rathnayaka et al., 2017, proposed two new calibration methods that use a specially designed, colored checkered board pattern and mathematical pose estimation approaches to calibrate a stereo camera setup with heterogeneous lenses, i.e., a wide-angle fish-eye lens and a narrow-angle lens [11]. One method calculates left and right transformation matrices for stereo calibration, while the other utilizes a planar homography relationship and Zhang’s calibration method to identify common object points in stereo images. Evaluations based on $\epsilon_{RP}$ and image rectification results showed both methods successfully overcome the challenges, with the planar homography approach yielding slightly more accurate results. Wang et al., 2018, proposed a non-iterative self-calibration algorithm to compute the extrinsic parameters of a rotating (yaw and pitch only) and a non-zooming camera [13]. The algorithm utilizes a single feature point to improve the calibration speed and Zhang’s method was employed in advance to calculate the intrinsic parameters of the camera. Although their results are slightly less accurate than those calibrated by Zhang’s method alone, their work demonstrates its potential for real-time applications where extremely high calibration accuracy is not required. Hu et al., 2020, introduced a calibration approach for binocular cameras that begins with Zhang’s technique to obtain an initial guess of the rotation and translation matrix [5]. This guess is then refined using singular value decomposition and solved via the Frobenius norm, and is further refined through maximum likelihood estimation, generating a new calculation for the relative position matrix of the binocular cameras. The Levenberg–Marquardt algorithm is employed for additional refinement. Their results show significant improvement over the traditional Bouget’s and Hartley’s algorithms. Liu et al., 2022, proposed a multi-camera calibration stereo calibration method focusing on the high-precision extraction of circular calibration pattern features during the calibration process [20]. The Franklin matrix, used for sub-pixel edge detection and the image moment method, was used to obtain the characteristic circle center, while the calibration was realized using Zhang’s method. Their work shows that calibration effects can be improved by focusing on the high-precision extraction of calibration features.

Calibration targets also play a significant role in camera calibration. Although using a checkered board pattern as the calibration target does have the advantages, such as simplicity, stability, and a generally higher accuracy than self-calibration, it has the downside of being susceptible to illumination and noise in corner feature extraction and matching. Circular patterns have been proposed to address the problem [21]. Recently, Wei et al., 2023, presented a camera calibration method using a circular calibration target [22]. Their method operates in two stages. The first stage involves extracting the centers of the ellipses and formulating the real concentric circle center projection equation by leveraging the cross-ratio invariance of collinear points. The second stage involves solving a system of linear equations to find the other center projection coordinates, taking advantage of the fact that the infinite lines passing through the centers are parallel. Unlike other circular calibration algorithms that directly fit ellipses to estimate the centers of circular feature points, their method not only corrects errors that arise from using the ellipse’s center but also significantly improves the accuracy and efficiency of the calibration process. The proposed method reduces the average $\epsilon_{RP}$ to 0.08 pixels and achieves real-world measurement accuracy of 0.03 mm, making it valuable for high-precision 3D measurement tasks. Recently, Zhang et al., 2023, proposed a flexible calibration method for large-range binocular vision systems for complex construction environments [23]. The calibration method consists of two stages. In stage 1, lenses are focused closer to the cameras, and non-overlapping fields of view (FOVs) are calibrated using virtual projection point pairs through Zhang’s method and nonlinear optimization. Fourteen sets of clear checkered board images are acquired for this purpose. Then, the lenses are focused on the measurement position in stage 2, where three sets of blurred three-phase shifted circular grating (PCG) images are taken. The final extrinsic parameters for the binocular camera are computed using state transformation matrices for calibration. The method has demonstrated robustness under varying target angles and calibration distances, with a mean $\epsilon_{RP}$ of only 0.0675 pixels and a relative measurement error of less than 1.75% in their experiment.

While Zhang’s method ensures accurate calibration outcomes when viewing a planar target from various angles, the selection of these differing orientations relies largely on empirical knowledge, which could potentially lead to variations in the calibration results [24]. Furthermore, it is often necessary to manually adjust the calibration object’s position, a process that introduces uncertainties due to instability and shaking, resulting in inconsistent positioning of the calibration plate across images captured by different cameras, and thus introducing inherent errors that directly affect the calibration process’s accuracy [15]. Moreover, the calibration procedure requires a significant amount of time and effort [13].

The present study seeks to address numerous key issues related to camera calibration in the process of capturing checkered board images. In particular, it aims to optimize this process by identifying the optimal distance and angle for calibration, consequently enhancing the calibration results. Different from the existing literatures, this study places emphasis on gaining a comprehensive understanding of the factors that affect $\epsilon_{RP}$ in the calibration process, specifically the roles of various displacement and orientation factors, and how they might be manipulated independently for better calibration efficiency. This leads to four principal research questions which revolve around understanding the impacts of distance factors (*D*, *H*, *V*) and orientation factors (*R*, *P*, *Y*) on $\epsilon_{RP}$, determining the effects of combined distance and orientation factors on $\epsilon_{RP}$, and exploring the feasibility and efficiency of conducting the calibration process separately for different factor groups. To address these, the research objectives include identifying the most influential distance and orientation factors, investigating the combinations of factors that minimize $\epsilon_{RP}$, and evaluating the efficiency of separately conducting the calibration process for different factor groups. The overarching goal is to develop more efficient and effective calibration procedures for various applications.

## 2. Methodology

### 2.1. Experiment Setup

Three different cameras are used in this study. The details of the cameras are presented in Table 1. However, only the calibration process of the ZED2i binocular camera [25] is presented in this study.

**Figure 1:**
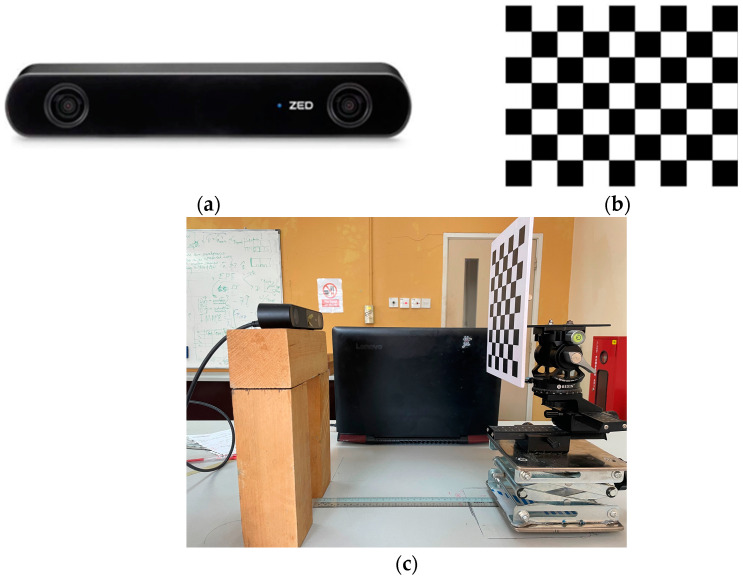
Calibration experiment. (**a**) ZED2i binocular camera [25]; (**b**) calibration checkered board; (**c**) experimental setup.

For camera calibration, a checkered board consisting of 2 cm alternating black and white squares arranged in a grid pattern of nine columns and seven rows is employed, as shown in Figure 1b. The checkered board is attached to the camera calibration rig as shown in Figure 1c. The Stereo Camera Calibration Toolbox for MATLAB [18] is used for calibration, where the inner corner points are detected using the Harris corner algorithm [26]. The checkered board size is fed to the algorithm and the actual number of detected corner points is 48. The high contrast between black and white aids in accurately detecting and localizing the inner corners of the squares, which are crucial for camera calibration algorithms.

**Figure 2:**
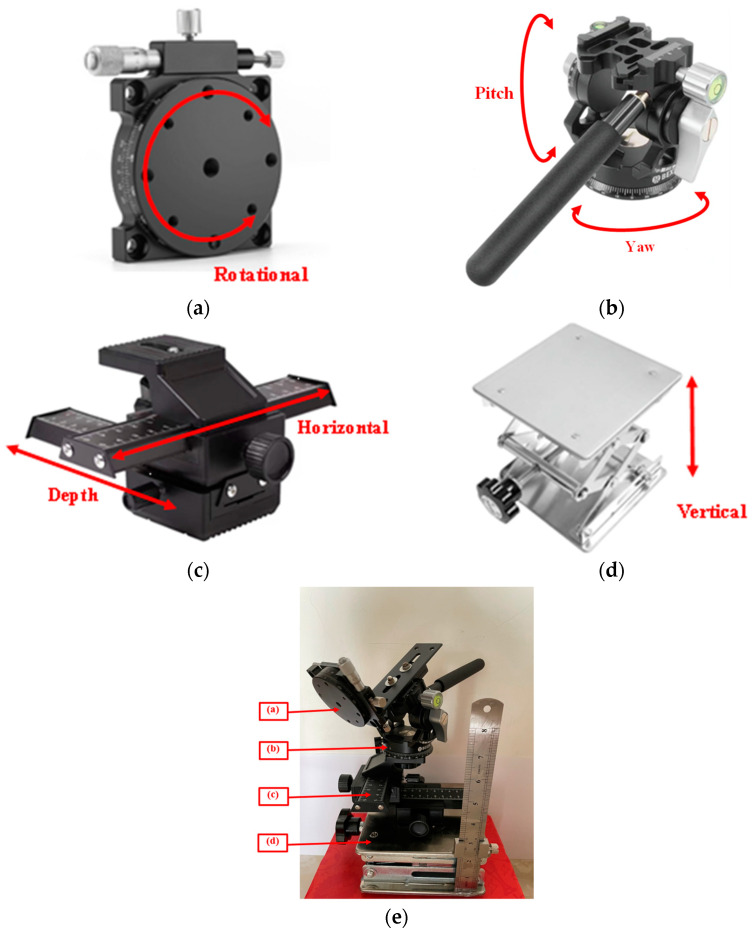
Components of the camera calibration rig. (**a**) RSP60R axis rotation stage; (**b**) LD2R dual panoramic gimbal; (**c**) two-way macro frame; (**d**) lift table; (**e**) complete assembly.

Since binocular camera calibration necessitates capturing images from various directions and angles, a camera calibration rig was designed to securely hold the checkered board, ensuring precise distances and orientations for calibration purposes. The components of the calibration rig and its final assembly are depicted in Figure 2. The rig comprises four main components: an LD2R dual panoramic gimbal for yaw (Y) and pitch (P) adjustments; a two-way macro frame for left and right (H) and depth, i.e., front and back (D) displacements; a lift table for vertical (V) movements; and an RSP60R axis rotation stage for rotation (R). These adjustments ensure that the checkered board is distributed across different quadrants and acquired from various positions and angles.

### 2.2. Zhang’s Camera Calibration

Zhang’s calibration method [16] was used in this study to perform the calibration task due its simplicity and robustness. Mathematically, given the point $P={\left(X_{w},\;Y_{w},\;Z_{w}\right)}^{T}$ represents the coordinates of each corner point on the checkered board in the world coordinate system, its homogeneous coordinate is $\tilde{P}=\left(X_{w},\;Y_{w},\;Z_{w},\;1\right)$. In the pixel coordinate system, this point is denoted by $m={\left[u,\;v\right]}^{T}$, while its homogeneous coordinate is $\tilde{p}={\left[u,\;v,\;1\right]}^{T}$. The relationship between the world coordinate and the image coordinate is described by the camera model, as follows:

(1) $$\lambda \tilde{p}=A\left[R|t\right]\tilde{P}$$

where λ is the proportionality coefficient and [R|t] and A are the extrinsic and intrinsic parameter matrices of the camera, respectively. By assuming that Z=0 for the planar board, the equation can be further simplified:

(2) $$\lambda \tilde{p}=A\left[r_{1}\;r_{2}\;t\right]\left(\begin{array}{c} X_{w}\\ Y_{w}\\ 1 \end{array}\right)=H\tilde{P}$$

where H is a 3×3 homography matrix, defined as follows:

(3) $$H=\left[h_{1}\;h_{2}\;h_{3}\right]=sA[r_{1}\;r_{2}\;t]$$

and s is a proportional factor. The homography matrix describes the transformation between two images of the same planar scene. Before calculating the homography matrix for camera calibration using a checkered board, it is necessary to configure the parameters for the world coordinates of the checkered board. This includes details such as the board’s overall size, the size of each square, and the number of detected corner points on the checkered board.

The fundamental constraints for the camera’s intrinsic parameters are as follows:

(4) $$\begin{array}{c} h_{1}^{T}A^{-T}A^{-1}h_{2}=0\\ h_{1}^{T}A^{-T}A^{-1}h_{1}=h_{2}^{T}A^{-T}A^{-1}h_{2}=1 \end{array}$$

By using the properties of the rotation matrix R, the unit orthogonal vectors $r_{1}$ and $r_{2}$ can be obtained. The camera intrinsic parameters can be determined by solving $A^{-T}A^{-1}$, using Equation (4).

**Figure 3:**
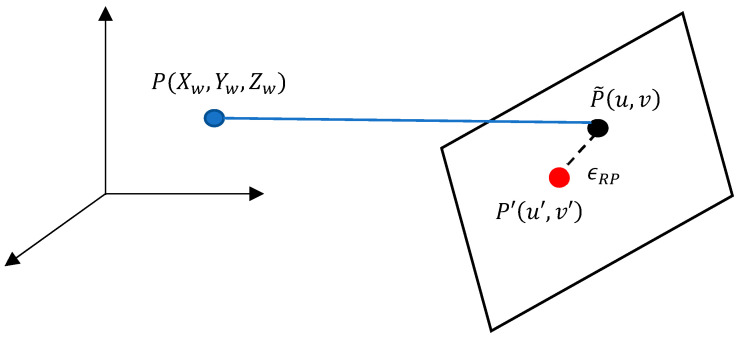
Schematic representation of reprojection error.

$\epsilon_{RP}$ is the error between the reprojected point, obtained by projecting the calibrated image point onto the camera coordinate system and its actual coordinate. This metric is used to assess the accuracy of camera calibration results in this study. As shown in Figure 3, after calibrating the camera using its intrinsic and extrinsic parameters, the point P is reprojected onto the image plane, resulting in a new point $P^{\prime }$, with pixel coordinates $\left(u^{\prime },\;v^{\prime }\right)$. The calibration $\epsilon_{RP}$ is calculated as the Euclidean distance between the reprojected pixel coordinates $\left(u^{\prime },\;v^{\prime }\right)$ and the actual calibrated pixel coordinates (u, v). The formula for the $\epsilon_{RP}$ is given by Equation (5).

(5) $$\epsilon_{RP}=\sqrt{{\left(u-u^{\prime }\right)}^{2}+{\left(v-v^{\prime }\right)}^{2}}$$

### 2.3. Design of Experiment (DoE)

**Figure 4:**
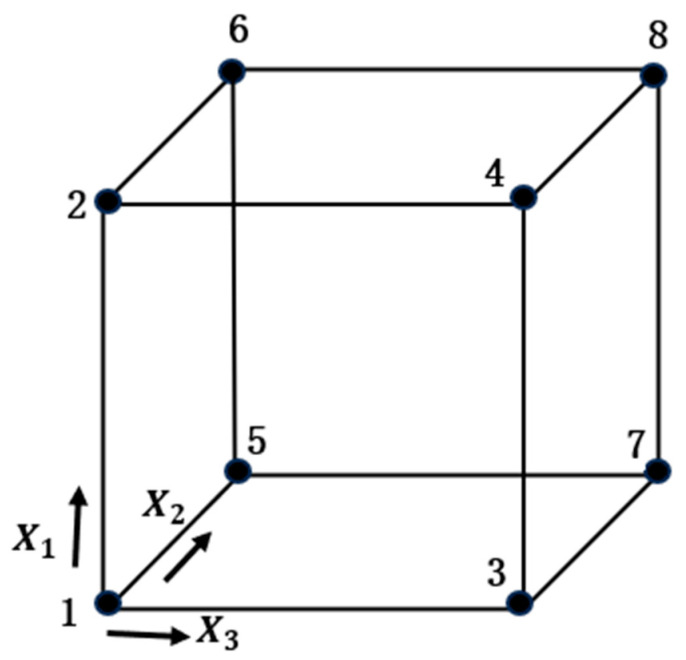
Representation of the $2^{3}$ design space [27].

A 2^k^ full factorial with replication design of experiment method [27] is employed to analyze the influence of each factor and their interactions on $\epsilon_{RP}$ in the camera calibration. A 2^k^ full-factorial DoE is a type of experimental design used to study the effect of k independent factors at 2 levels each (i.e., the low and high bounds) on a response variable. This allows for all possible combinations of the factors at all levels to be investigated, providing a comprehensive view of the entire experimental space. Figure 4 depicts the k=3 factor design space, where each corner point of the cube represents the combination of the factors $X_{1},\;X_{2},\;$ and $X_{3}$. With replication, i.e., by repeating the entire experiment with more than one operating condition, the errors or uncertainties introduced by the other variables can be estimated and the significance and interactions of the independent factors can be revealed.

The factors involved in this study can be divided into two categories: orientation factors (*R*, *P*, and *Y*) and displacement factors (*D*, *V*, *H*). Given that Zhang’s method requires a minimum of three sets of images for calibration, specific variations must be incorporated into each experimental set. Consequently, the calibration experiment must be designed to exclude some factors to accommodate these variations. In this study, the experiments are configured as follows: Method A employs displacement factors for calibration while introducing variations in orientation. Method B utilizes orientation factors for calibration and introduces variations in displacement. Method C incorporates the two most significant factors from both orientation and displacement categories while introducing variations to the remaining factors.

**Figure 5:**
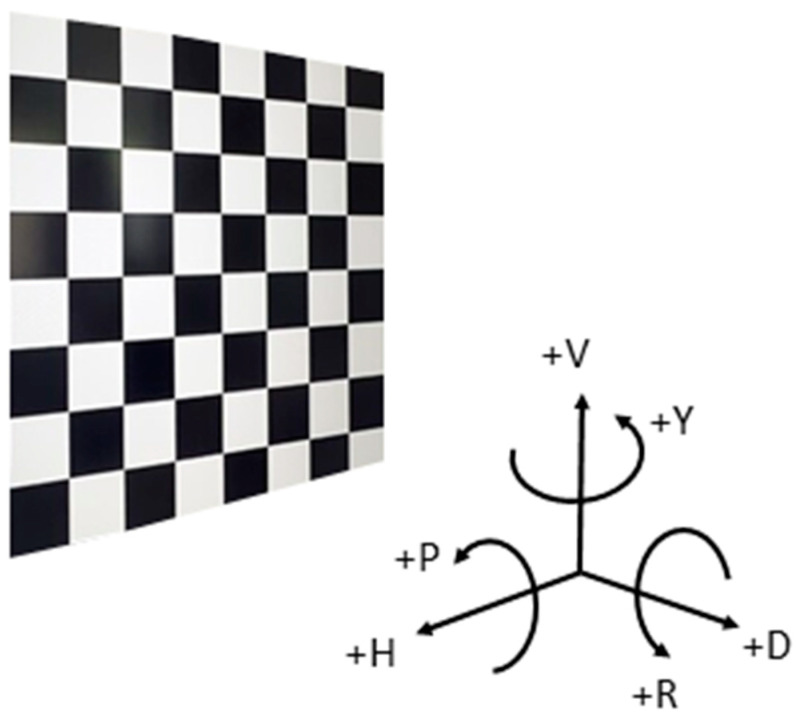
Sign convention.

The bounds for displacement and orientation factors are set at ±3 cm and ±10°, respectively. These displacement boundaries are designed to ensure that the checkered board stays within the field of view (FOV) of the camera, considering the minimum distance between the board and the camera. Upward, leftward, and forward displacements are categorized as positive, while downward, rightward, and backward displacements are considered negative. As for the orientation limits, the specific values are chosen based on experimental data to guarantee that all corners of the images captured by the camera can be detected using Zhang’s method. Positive orientations are defined as up pitch, right yaw, and left roll, whereas negative rotations are classified as down pitch, left yaw, and right roll. The convention for sign determination is presented in Figure 5.

The experiment process starts by generating the 2^k^ full-factorial design points using the factors of interest. For methods A and B, each involves a factor group of three factors with two levels, resulting in a total of $2^{3}$ = 8 combinations for each experiment. On the other hand, for method C, the two most important factors from each factor group are selected to construct the design points, resulting in $2^{4}$ = 16 combinations.

**Figure 6:**
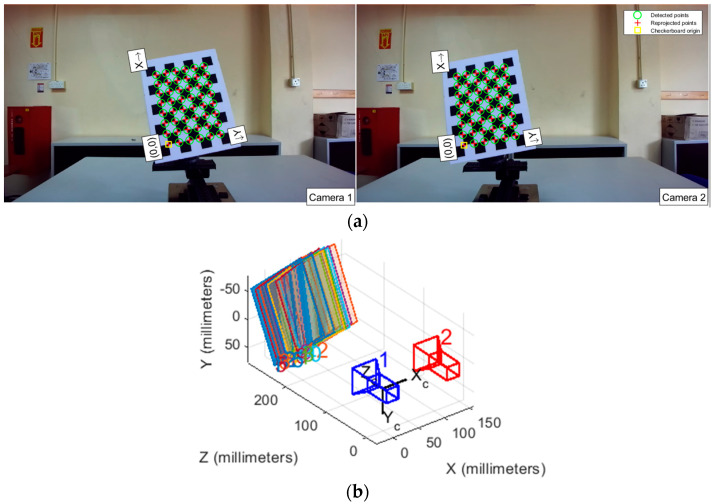
Calibration processes: (**a**) calibration board corner detection; (**b**) 20 sets of calibration images and the positions of the binocular camera.

Then, Latin Hypercube Sampling (LHS) is used to select 20 points from the remaining factors to introduce variations for Zhang’s method. LHS is used to ensure that the variations introduced by the remaining factors are well distributed within the multidimensional sampling space, allowing them to be treated as random variables in the analysis. The calibration process is presented in Figure 6.

The interaction effects response model, Y, is built to describe the relationship between the factors and the response variable, i.e., $\epsilon_{RP}$:

(6) $$Y=\beta_{0}+\beta_{1}X_{1}+\beta_{2}X_{2}+\cdots +\beta_{k}X_{k}+\sum_{i<j}\beta_{ij}X_{i}X_{j}+\varepsilon$$

In Equation (6), $\beta_{0}$ is the intercept term, which is the baseline level of the response Y when all factors $X_{1},X_{2},\cdots ,X_{k}$ are zero. The terms $\beta_{1},\;\beta_{2},\cdots ,\beta_{k}$ are the model coefficients for the main effects of the factors $X_{1},X_{2},\cdots ,X_{k}$, respectively. Each $\beta_{i}$ represents the change in Y for a unit change in $X_{i}$ when all other factors are held constant. A high absolute value of $\beta_{i}$ indicates that its corresponding factor $X_{i}$ has a strong influence on Y, and vice versa. The term $\sum_{i<j}\beta_{ij}X_{i}X_{j}$ denotes the interaction effects between the factors $X_{i}$ and $X_{j}$. Finally, ε is the random error term, describing the uncertainty or unexplained variability in the model.

Bayesian Information Criterion (BIC) is used to quantify the trade-off between the goodness of fit of the response model and its complexity [28]:

(7) $$BIC=k\ln\left(n\right)-2\ln\left(\hat{L}\right)$$

where $\hat{L}$ is the maximized value of the likelihood function of the response model, n is the sample size, and k is the number of parameters estimated by the model. The term $kln\left(n\right)$ is the penalty function for model complexity, to discourage overfitting by keeping the number of k low. The term $-2\ln(\hat{L})$ is the measure of the goodness of fit of the model to the data, where smaller values indicate a better model fit. In model selection, model with the lowest BIC is considered the best.

**Figure 7:**
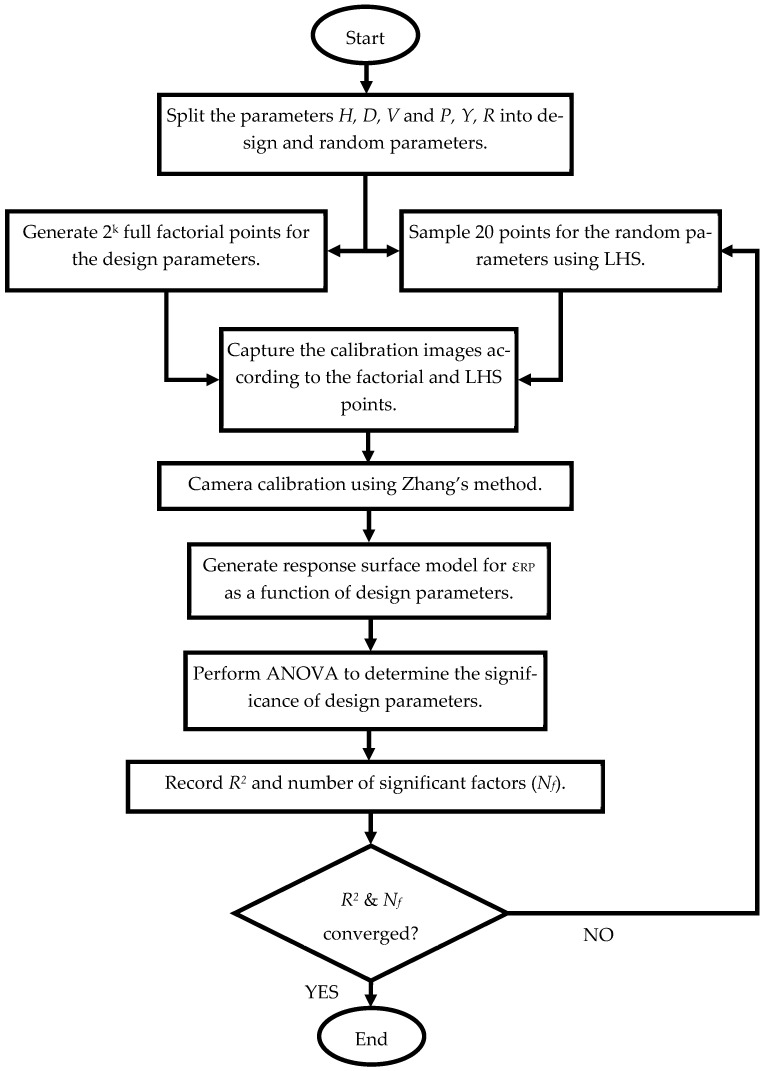
Experiment flow chart.

Analysis of variance (ANOVA) is used to determine which factors are statistically significant in the response. The *R^2^*, *R^2^*-adjusted, *R^2^*-predicted, and the number of significant factors ($N_{f}$), are used to judge whether additional replication is needed to yield a stable response. In this study, MINITAB [29] is used to generate the factorial points and perform the DoE analysis. The experimental procedure is summarized in Figure 7.

## 3. Results and Discussion

### 3.1. Method A: Calibration with Displacement Factors

**Figure 8:**
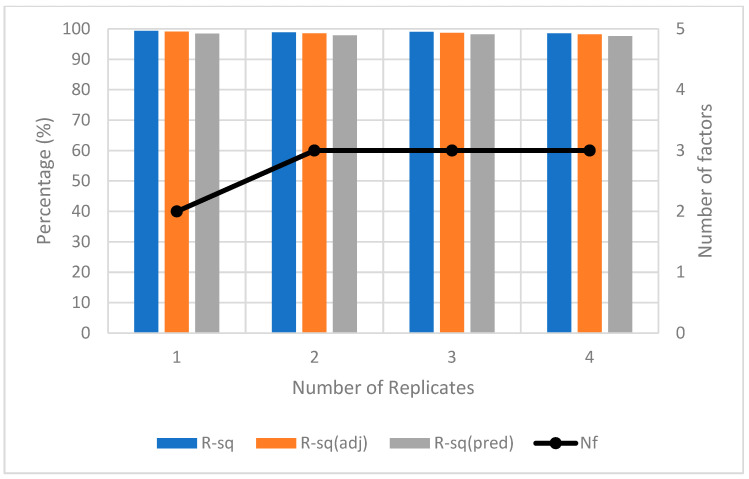
Convergence plot for method A for ZED2i camera.

Method A is designed to calibrate with displacement factors, i.e., *D*, *H*, and *V*, on $\epsilon_{RP}$. LHS was employed to randomly sample 20 points within the range of ±10° for 3 orientation factors, *R*, *P*, and *Y*. The experiment is conducted by adding one DoE replicate at a time until the $R^{2},$ adjusted $R^{2}$, predicted $R^{2}$ values, and the number of significant factors ($N_{f}$) of the response model in the successive replicate are consistent. As shown in Figure 8, only three replicates are needed to reach convergence. An additional replicate was used to verify the results. The total number of photos taken to yield convergence is 3 replicates × 20 LHS samples × 8 factorial points = 480. The resultant hierarchical $\epsilon_{RP}$ response model in uncoded units is presented in Equation (8).

(8) $$\epsilon_{RP}=0.085578+0.003441\cdot D+0.000211\cdot H+0.000341\cdot V$$

**Figure 9:**
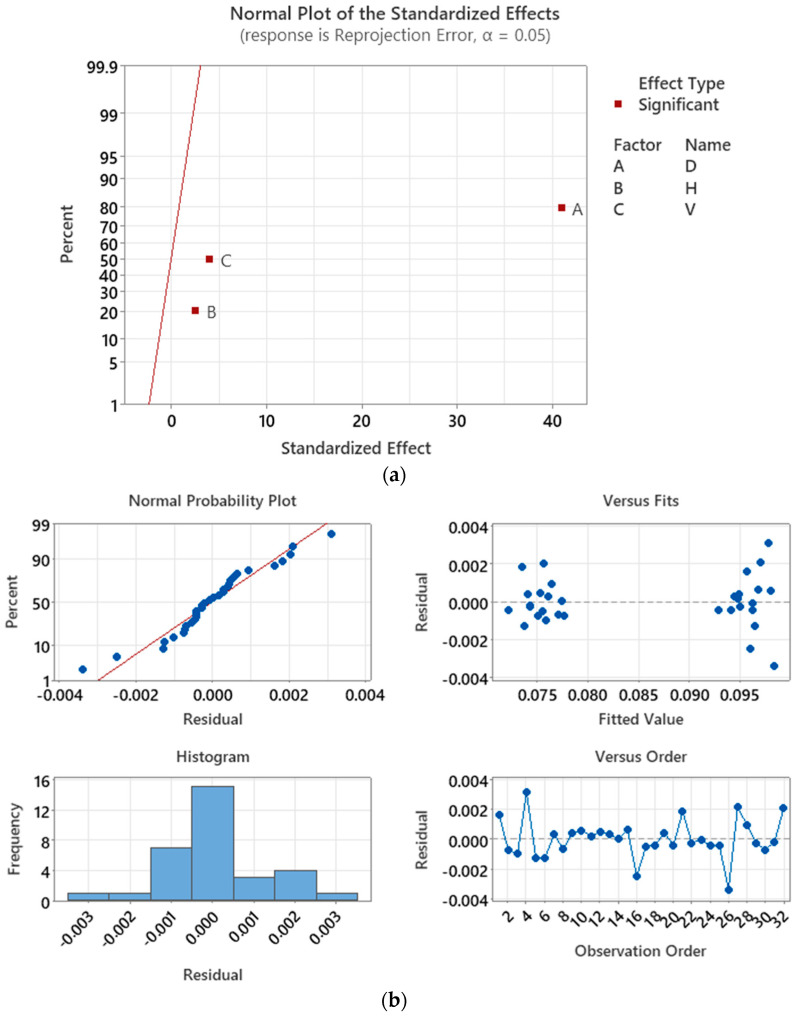
Calibration of ZED2i camera with method A. (**a**) Normal plot of the standardized effects; (**b**) $\epsilon_{RP}$ residual plot.

The model is reduced to a linear regression. Within the limits of the factors, the minimum $\epsilon_{RP}$ can be achieved by minimizing the values of the factors. Figure 9a displays the normal plot of the standardized effects of the response model, which confirms that only the three main factors are significant for $\epsilon_{RP}$. Among these factors, *D* exhibits the most influence on $\epsilon_{RP}$ for method A, followed by *V* and *H*. Additionally, the normal plot reveals that all three factors have positive effects on the $\epsilon_{RP}$, indicating that an increase in the factor’s level will result in an increase in $\epsilon_{RP}$.

The residual plot in Figure 9b confirms that the residuals of the model follow a normal distribution with zero mean, indicating that the model effectively accounts for the systematic variation in the data, leaving only random variation unexplained. The factorial point where *D* = *H* = *V* = −3 cm exhibits the lowest $\epsilon_{RP}$, with a value of 0.0716 pixels. This result aligns with the predictions made by the linear regression model, where $\epsilon_{RP}$ = 0.0733 is minimum for this combination.

### 3.2. Method B: Calibration with Orientation Factors

**Figure 10:**
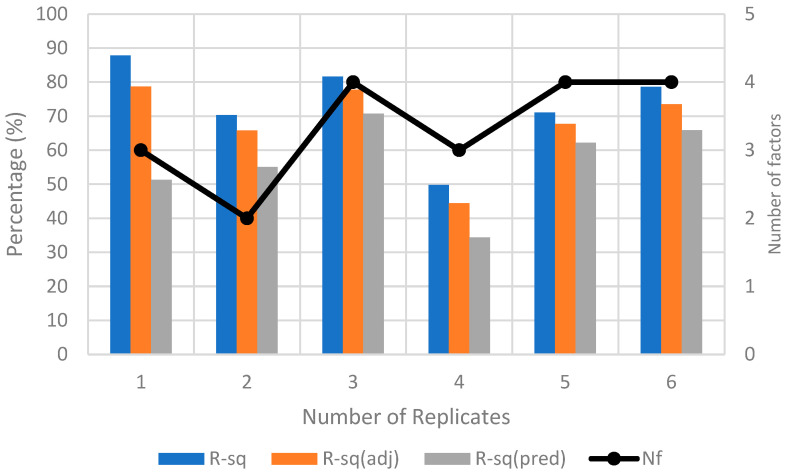
Convergence plot for method B for ZED2i camera.

Method B calibrates with the three orientation factors, i.e., *R*, *P*, and *Y* on $\epsilon_{RP}$. LHS was employed to randomly sample 20 points within the range of ±3 cm for 3 displacement factors, *D*, *H*, and *V*. The calibration is conducted by adding one DoE replicate at a time until the $R^{2}$, adjusted $R^{2}$, predicted $R^{2}$ values, and the number of significant factors ($N_{f}$) of the response model in the successive replicate are consistent. As shown in Figure 10, six replicates are needed to reach convergence. The total calibration images acquired is 960. The resultant hierarchical $\epsilon_{RP}$ response model in uncoded units is presented in Equation (9).

(9) $$\epsilon_{RP}=0.088156+0.000100\cdot R-0.000166\cdot P-0.000056\cdot Y+0.000014\cdot R\cdot P$$

**Figure 11:**
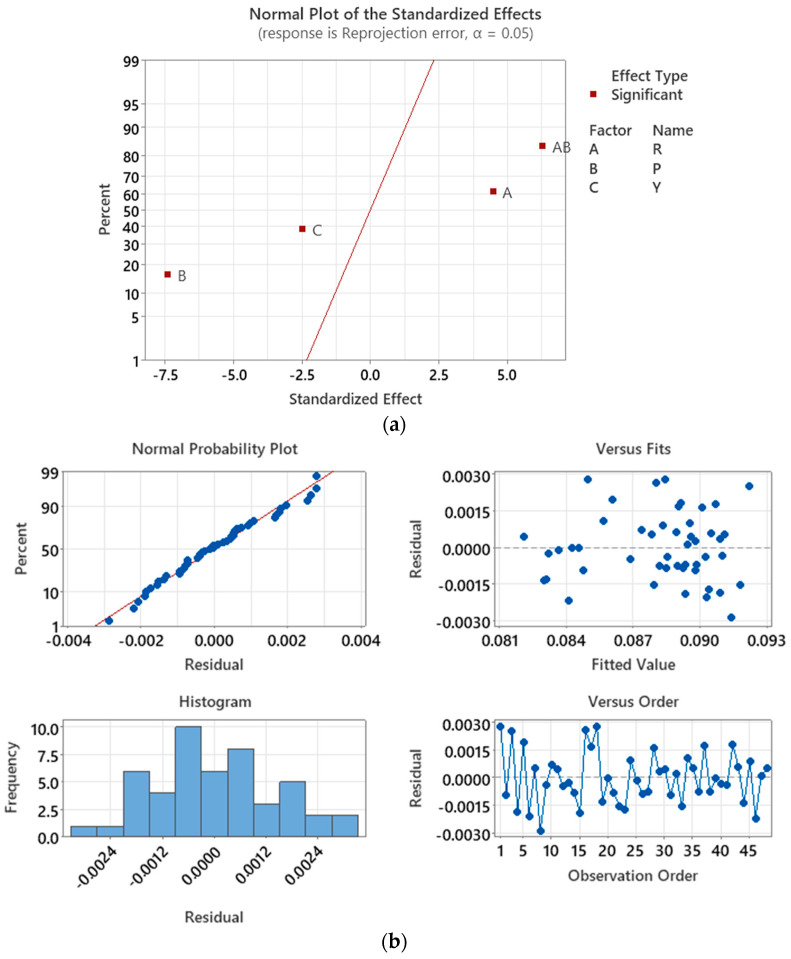
Calibration of ZED2i camera with method B. (**a**) Normal plot of the standardized effects; (**b**) $\epsilon_{RP}$ residual plot.

Within the limits of the factors, the minimum $\epsilon_{RP}$ can be achieved by minimizing *R* and maximizing *P* and *Y*. Figure 11a displays the normal plot of the standardized effects of the response model, which confirms that the three main orientation factors and an additional R⋅P interaction factor are significant for $\epsilon_{RP}$. Among these factors, *P* exhibits the most influence on $\epsilon_{RP}$ for method B, while the R⋅P interaction factor ranked the second, followed by *R* and *Y*. Additionally, factors *P* and *Y* have are inversely correlated with $\epsilon_{RP}$, suggesting an increase in the factor’s level will result in a decrease in $\epsilon_{RP}.$ In contrast, an increase in the level of factor *R* and R⋅P interaction will lead to an increase in $\epsilon_{RP}.$

The residual plot in Figure 11b confirms that the residuals of the model follow a normal distribution with zero mean, indicating that the model effectively accounts for the systematic variation in the data, leaving only random variation unexplained. The factorial point is where *R =* −10° and *P* = *Y* = 10° exhibits the lowest $\epsilon_{RP}$, with a value of 0.0825 pixels. This result aligns with the predictions made by the linear regression model, where $\epsilon_{RP}$ = 0.0835 is minimum for this combination.

### 3.3. Method C: Calibration with Displacement and Orientation Factors

**Figure 12:**
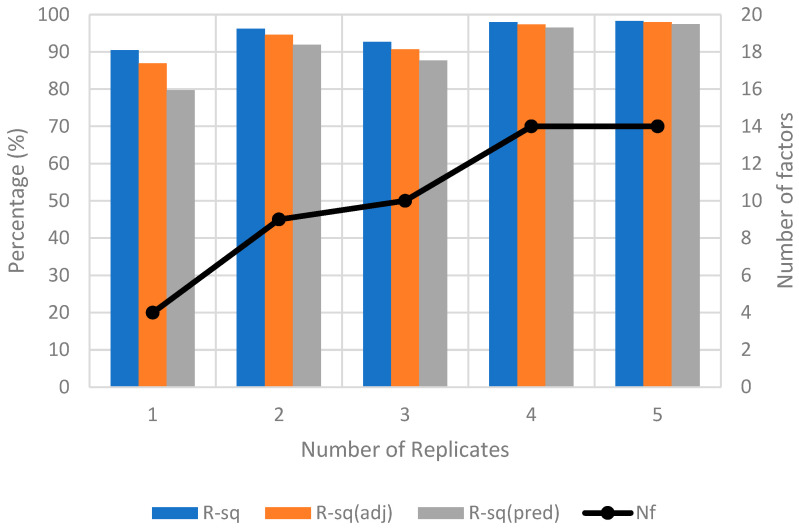
Convergence plot for experiment method C.

Differently from methods A and B, which calibrate each factor group on $\epsilon_{RP}$ separately, method C calibrates the combined displacement and orientation factors concurrently. The two most prominent factors from each factor group are selected. LHS was employed to randomly sample 20 points from the remaining factors withing their respective range. The experiment is conducted by adding one DoE replicate at a time until the $R^{2}$, adjusted $R^{2}$, predicted $R^{2}$ values, and $N_{f}$ of the response model are consistent in the successive replication. As shown in Figure 12, 5 replicates are needed to reach convergence, resulting in a total of 1600 calibration images acquired. The non-hierarchical $\epsilon_{RP}$ response model in coded units is presented in Equation (10).

(10) $$\begin{array}{ll} \epsilon_{RP}=0.085931 & +0.003511\cdot R-0.006806\cdot P+0.011506\cdot D-0.001726\cdot R\cdot P\\ & +0.001441\;R\cdot D-0.001751\cdot R\cdot V-0.001671\cdot P\cdot D\\ & +0.003841\cdot P\cdot V-0.001091\cdot D\cdot V-0.000886\cdot R\cdot P\cdot D\\ & +0.001411\cdot R\cdot P\cdot V-0.000526\cdot R\cdot D\cdot V+0.001251\cdot P\cdot D\cdot V\\ & +0.000826\cdot R\cdot P\cdot D\cdot V \end{array}$$

**Figure 13:**
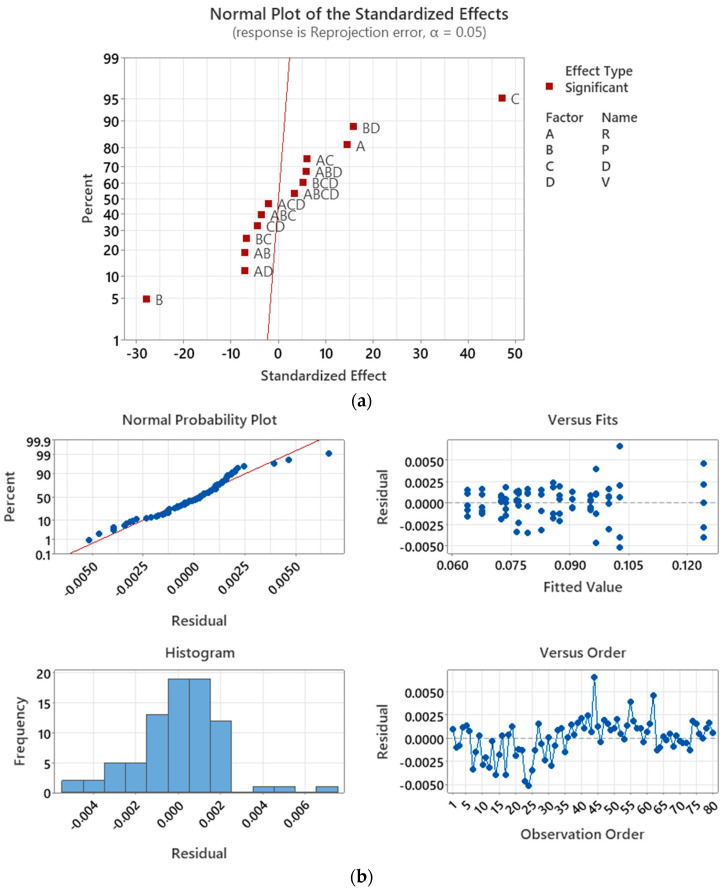
Calibration of ZED2i camera with method C. (**a**) Normal plot of the standardized effects; (**b**) $\epsilon_{RP}$ residual plot.

Figure 13a presents the normal plot of the standardized effects of $\epsilon_{RP}$. Two main orientation factors *R* and *P* and a displacement factor *D* are significant for $\epsilon_{RP}$. Factors *P* and *D* are positively correlated to $\epsilon_{RP}$, while R is negatively correlated to $\epsilon_{RP}$. Additionally, eleven interaction factors appear to be significantly influential on $\epsilon_{RP}$ for method C.

The residual plot in Figure 13b confirms that the residuals of the model follow a normal distribution with zero mean, indicating that the model effectively accounts for the systematic variation in the data, leaving only random variation unexplained. The minimum $\epsilon_{RP}$ obtained from Equation (10) is 0.0622 pixels, and the corresponding *R*, *P*, *D*, and *V* values are −10°, +10°, −3 cm, and −3 cm, respectively. This result aligns with the measurement, where $\epsilon_{RP}$ = 0.0638 pixles is minimum for this combination. Interestingly, the *R*, *P*, *D*, and *V* values that yield the minimum $\epsilon_{RP}$ are also consistent with the values for methods A and B.

### 3.4. Comparisons of Methods A, B, and C

Two additional cameras, D435i and HBVCAM-W202011HD V33, are used to assess the calibration performance of methods A, B, and C. For method A, which used only displacement factors for camera calibration, it was found that factors *D*, *H*, and *V* are linearly correlated to $\epsilon_{RP}$ for all three cameras involved in this study. Factor *D* is the most influential factor on $\epsilon_{RP}$ for all cameras, followed by *V* and *H* for the ZED2i camera, and H and V for both D435i and HBVCAM. However, a closer examination on Figure 9a reveals that both *H* and *V* factors are equally important. For method B, which calibrates using the orientation factors, it was observed that factors R and P are more influential than Y on $\epsilon_{RP}$ for all cameras. Therefore, factors *D* and *H* or *V* from the distance group and factors *P* and *Y* from the orientation group are recommended for calibration with method C.

Table 2 compares the calibration results for ZED2i camera using methods A, B, and C. It is evident that combining the two most significant factors, which yield the minimum $\epsilon_{RP}$ for methods A and B, respectively, will also result in the minimum $\epsilon_{RP}$ for method C. The results also show that a total of 1600 images are needed for calibration with method C, while methods A and B require only 1440 images in total, about 10% less compared to method C.

The focal lengths in the x and y directions ($f_{x}$, $f_{y}$), the principal point ($c_{x}$, $c_{y}$), and the radial distortion matrix ($k_{1},\;k_{2},\;k_{3}$) of the ZED2i camera are presented in Table 3. The tangential distortion matrix ($p_{1},\;p_{2}$) is set to zero in this study.

**Figure 14:**
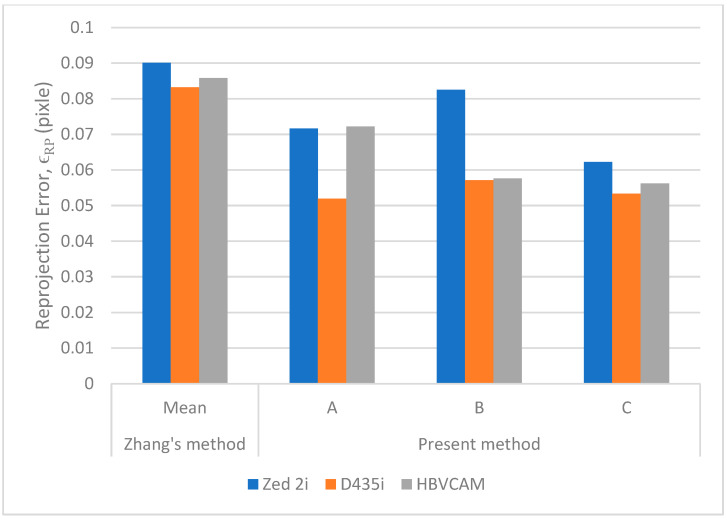
Calibration performance of methods A, B, and C on 3 different cameras.

Zhang’s method is used to benchmark the performance of the calibration results. To improve the reliability of Zhang’s calibration, the calibration is repeated five times, each with 20 sets of images of the randomly positioned calibration target. The average $\epsilon_{RP}$ values obtained are 0.0901, 0.0832, and 0.0858 pixels for cameras ZED2i, D435i, and HBVCAM-W202011HD V33, respectively. The results are presented in Figure 14.

Figure 14 shows that methods A, B, and C are able to reduce $\epsilon_{RP}$. For ZED2i, D435i, and HBVCAM cameras, method A is able to reduce their respective $\epsilon_{RP}$ by 20%, 38%, and 16% compared to Zhang’s method. For method B, the $\epsilon_{RP}$ reductions for the three cameras are 8%, 31%, and 33%. The average $\epsilon_{RP}$ reduction by methods A and B are 25% and 24%, respectively, suggesting that both methods are equally effective. On the other hand, method C can produce a more consistent calibration result. The $\epsilon_{RP}$ reductions for the three cameras are 31%, 36%, and 34%, respectively.

**Figure 15:**
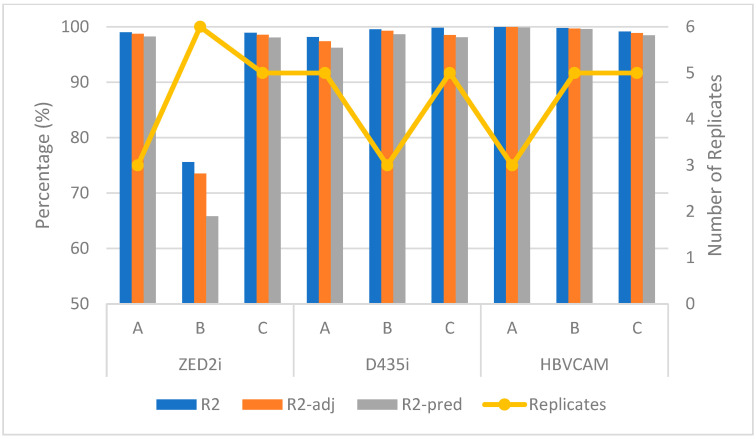
Modeling performance of methods A, B, and C on 3 different cameras.

Figure 15 shows the modeling performance for the three cameras using methods A, B, and C. The results indicate that the proposed 2^k^ full-factorial DoE is effective in modeling $\epsilon_{RP}$, as most of the models are able to achieve the $R^{2}$, adjusted $R^{2}$, and predicted $R^{2}$ values of more than 95%, except for method B for the ZED2i camera, where its $R^{2}$, adjusted $R^{2}$, and predicted $R^{2}$ values are less than 75%. A closer examination of the $\epsilon_{RP}$ residuals for methods A, B, and C (see Figure 9b, Figure 11b and Figure 13b) reveals that that data for method B (Figure 11b) contain high variability. More replications can be carried out to improve its calibration accuracy in this case. However, method B is still able to capture the underlying significant factors and their interactions for ZED2i camera.

Interestingly, the factorial point with *D* = *H* = *V* = −3 cm exhibited the lowest $\epsilon_{RP}$ for all cameras calibrated with method A. Similarly, the factorial point with *R* = −10° and *P* = *Y* = 10° resulted in the minimum $\epsilon_{RP}$ for all cameras calibrated with method B. For method C, the minimum $\epsilon_{RP}$, obtained by considering the factorial point with *R* = −10°, *P* = 10°, and *D = V* = −3 cm, is consistent with the results from methods A and B. Since the baseline of each camera was used to determine the default depth distance for calibration to ensure the checkered board is within its FOV, it has no impact on $\epsilon_{RP}$.

## 4. Conclusions

This paper adopts Zhang’s camera calibration method, combined with the 2^k^ full-factorial DoE and LHS methods, to minimize $\epsilon_{RP}$ in camera calibration. In general, the results of the experiments conducted for methods A, B, and C provide valuable insights into the factors affecting $\epsilon_{RP}$ in the calibration process.

For method A, which used only displacement factors for camera calibration, it was found that factors *D*, *H*, and *V* are linearly correlated to $\epsilon_{RP}$ for all three cameras involved in this study. Factor *D* was found to be the most influential factor on $\epsilon_{RP}$ for all cameras, followed by V and H for the ZED2i camera, and H and V for the D435i and HBVCAM cameras. However, a closer examination of Figure 9a reveals that both the H and V factors are equally important. The factorial point with *D* = *H* = *V* = −3 cm exhibited the lowest $\epsilon_{RP}$, confirming the predictions made by the linear regression model. The experiment demonstrated that by minimizing the values of the displacement factors, $\epsilon_{RP}$ can be reduced by 25% on average compared to Zhang’s method.

On the other hand, for method B, which calibrates using the orientation factors, it was observed that factors R and P are more influential than Y on $\epsilon_{RP}$ for all cameras. The factorial point with *R* = −10° and *P* = *Y* = 10° resulted in the minimum $\epsilon_{RP}$. The findings were consistent with the predictions made by the response models, highlighting the importance of controlling the orientation factors to minimize $\epsilon_{RP}$. On average, method B can reduce the $\epsilon_{RP}$ by 24% compared to Zhang’s method.

For method C, where two of the most influential factors from distance and orientation factors were selected for calibration, the minimum $\epsilon_{RP}$ was obtained at the factorial point with *R* = −10°, *P* = 10°, and *D* = *V* = −3 cm; this is consistent with the results from methods A and B. Although the method requires 10% more images compared to methods A and B combined, method C and produce a consistent minimum $\epsilon_{RP}$ with a reduction of more than 30% compared to Zhang’s method.

Comparing the results of methods A, B, and C, it is evident that combining the two most significant factors from methods A and B would also result in the minimum $\epsilon_{RP}$ for method C. Specifically, the combination of orientation factors *R* and *P* and displacement factors *D* and *V* or *H* can achieve the minimum $\epsilon_{RP}$ value for method C. Furthermore, it was found that the calibration process could be conducted separately and more efficiently by using the two factor groups independently to achieve more than 20% improvement in $\epsilon_{RP}$ compared to Zhang’s method alone. This approach required fewer calibration images, about 70% and 40% fewer for methods A and B, respectively, compared to method C. For applications where a lower value of $\epsilon_{RP}$ is required, method C is recommended.

Systematic camera calibration plays an important role in 3D reconstruction as it greatly influences the accuracy and reliability of the resulting 3D models. Accurate calibration ensures the precise determination of both the intrinsic and extrinsic parameters of a camera. This precision enables the correct mapping of 2D image data to real-world 3D coordinates. Optimizing camera calibration enhances the geometric fidelity of 3D reconstruction, paving the way for a wide range of applications in fields like computer vision, robotics, and augmented reality. Thus, it serves as the cornerstone underpinning the entire process of translating 2D images into accurate 3D reconstructions.

Overall, these findings contribute to a better understanding of the factors affecting $\epsilon_{RP}$ in calibration processes, enabling more efficient and effective calibration procedures in various applications. The proposed methods can be used to improve the calibration accuracy for stereo cameras for the applications in object detection and ranging. However, the proposed systematic camera calibration experiment involves a substantial amount of repetitive testing and the capture of numerous calibration checkered board photos, which consumes a significant amount of manpower and time resources. Additionally, camera distortions have a pronounced impact on camera calibration as they alter the geometric properties of camera imaging, subsequently affecting the precise mapping relationship between pixel coordinates and real-world coordinates. These two limiting factors will be subjected to in-depth exploration and discussion in future research.

## Figures and Tables

**Table 1:** Specifications of the three cameras used in the present study.

Specifications	ZED2i	D435i	HBVCAM-W202011HD V33
Baseline	120 mm	50 mm	61 mm
FOV	110° (H) × 70° (V) × 120° (D)	87° (H) × 58° (V) × 95° (D)	105°
Depth range	0.2–20 m	0.2–10 m	0.3 m-N/A

**Table 2:** Comparison of ZED2i camera calibration using methods A, B, and C.

Method	A	B	C
D (cm)	−3	LHS	−3
H (cm)	−3	LHS	LHS
V (cm)	−3	LHS	−3
R (°)	LHS	−10	−10
P (°)	LHS	10	10
Y (°)	LHS	10	LHS
No. of replicates	3	6	5
No. of interactions	0	1	10
No. of images taken	480	960	1600
$\epsilon_{RP}$ (pixel)	0.0716	0.0825	0.0638
Baseline (mm)	119.07	118.76	117.56

**Table 3:** The intrinsic parameters of ZED2i camera using methods A, B, and C.

Intrinsic Parameters		Method		
		A	B	C
Left camera	Focal length (pixel)	(534.00, 534.61)	(449.54, 494.71)	(515.16, 512.36)
	Principal point (pixel)	(617.36, 351.94)	(591.02, 351.96)	(615.94, 363.29)
	Radial distortion	(−0.11, 0.10, −0.04)	(−0.11, 0.08, −0.03)	(−0.12, 0.10, 0.00)
Right camera	Focal length (pixel)	(533.54, 533.84)	(500.57, 496.92)	(514.77, 515.49)
	Principal point (pixel)	(640.20, 360.92)	(500.57, 496.92)	(640.90, 369.25)
	Radial distortion	(−0.13, 0.14, −0.07)	(−0.12, 0.14, −0.08)	(−0.12, 0.10, −0.02)

## Data Availability

The data presented in this study are available on request from the corresponding author.

## References

[1] Muhovič J., Perš J. (2020). Correcting Decalibration of Stereo Cameras in Self-Driving Vehicles. Sensors.

[2] Feng M., Liu Y., Jiang P., Wang J. (2020). Object Detection and Localization Based on Binocular Vision for Autonomous Vehicles. J. Phys. Conf. Ser..

[3] Zhang M., Cai W., Xie Q., Xu S. (2022). Binocular-Vision-Based Obstacle Avoidance Design and Experiments Verification for Underwater Quadrocopter Vehicle. J. Mar. Sci. Eng..

[4] Yu X., Fan Z., Wan H., He Y., Du J., Li N., Yuan Z., Xiao G. (2019). Positioning, Navigation, and Book Accessing/Returning in an Autonomous Library Robot Using Integrated Binocular Vision and QR Code Identification Systems. Sensors.

[5] Hu G., Zhou Z., Cao J., Huang H. (2020). Highly Accurate 3D Reconstruction Based on a Precise and Robust Binocular Camera Calibration Method. IET Image Process..

[6] Hao Y.N., Tan Y.C., Tai V.C., Zhang X.D., Wei E.P., Ng S.C. (2022). Review of Key Technologies for Warehouse 3D Reconstruction. J. Mech. Eng. Sci..

[7] Zhong L., Qin J., Yang X., Zhang X., Shang Y., Zhang H., Yu Q. (2021). An Accurate Linear Method for 3D Line Reconstruction for Binocular or Multiple View Stereo Vision. Sensors.

[8] Zhou G., Sun X., Dong Q., Cao S., Li M. (2020). Research on Camera Calibration Method for Visual Inspection of Excavator Working Object. J. Phys. Conf. Ser..

[9] Chen C., Shen P. (2023). Research on Crack Width Measurement Based on Binocular Vision and Improved DeeplabV3+. Appl. Sci..

[10] Zhang B., Zhu D. (2021). Improved Camera Calibration Method and Accuracy Analysis for Binocular Vision. Int. J. Pattern Recognit. Artif. Intell..

[11] Rathnayaka P., Baek S.-H., Park S.-Y. (2017). An Efficient Calibration Method for a Stereo Camera System with Heterogeneous Lenses Using an Embedded Checkerboard Pattern. J. Sens..

[12] Yin H., Ma Z., Zhong M., Wu K., Wei Y., Guo J., Huang B. (2020). SLAM-Based Self-Calibration of a Binocular Stereo Vision Rig in Real-Time. Sensors.

[13] Wang Y., Wang X., Wan Z., Zhang J. (2018). A Method for Extrinsic Parameter Calibration of Rotating Binocular Stereo Vision Using a Single Feature Point. Sensors.

[14] Su Z., Lu L., Dong S., Yang F., He X. (2019). Auto-Calibration and Real-Time External Parameter Correction for Stereo Digital Image Correlation. Opt. Lasers Eng..

[15] Yin Z., Ren X., Du Y., Yuan F., He X., Yang F. (2022). Binocular Camera Calibration Based on Timing Correction. Appl. Opt..

[16] Zhang Z. (2000). A Flexible New Technique for Camera Calibration. IEEE Trans. Pattern Anal. Mach. Intell..

[17] Tsai R.Y. (1987). A Versatile Camera Calibration Techniaue for High-Accuracy 3D Machine Vision Metrology Using Off-the-Shelf TV Cameras and Lenses. IEEE J. Robot. Autom..

[18] Bouguet J.-Y. Camera Calibration Toolbox for Matlab (1.0) 2022. https://data.caltech.edu/records/jx9cx-fdh55.

[19] Heikkila J., Silven O. A Four-Step Camera Calibration Procedure with Implicit Image Correction. Proceedings of the Proceedings of IEEE Computer Society Conference on Computer Vision and Pattern Recognition.

[20] Liu X., Tian J., Kuang H., Ma X. (2022). A Stereo Calibration Method of Multi-Camera Based on Circular Calibration Board. Electronics.

[21] Liang S., Zhao Y. (2020). Camera Calibration Based on the Common Pole-Polar Properties between Two Coplanar Circles with Various Positions. Appl. Opt..

[22] Wei L., Zhang G., Huo J., Xue M. (2023). Novel Camera Calibration Method Based on Invariance of Collinear Points and Pole–Polar Constraint. J. Syst. Eng. Electron..

[23] Zhang H., Huo J., Yang F., Han J., Guo D. (2023). A Flexible Calibration Method for Large-Range Binocular Vision System Based on State Transformation. Opt. Laser Technol..

[24] Yin Y., Zhu H., Yang P., Yang Z., Liu K., Fu H. (2022). Robust and Accuracy Calibration Method for a Binocular Camera Using a Coding Planar Target. Opt. Express.

[25] ZED 2i—Industrial AI Stereo Camera. https://www.stereolabs.com/zed-2i/.

[26] Harris C., Stephens M. (1988). A Combined Corner and Edge Detector. Proceedings of the Alvey Vision Conference 1988.

[27] Das A.K., Dewanjee S. (2018). Optimization of Extraction Using Mathematical Models and Computation. Computational Phytochemistry.

[28] Konishi S., Kitagawa G. (2008). Bayesian Information Criteria. Information Criteria and Statistical Modeling.

[29] Minitab 2010. www.minitab.com.

